# Using informal caregivers’ experience data to inform quality improvement in healthcare settings: a mixed-methods systematic review

**DOI:** 10.1186/s12912-026-05065-1

**Published:** 2026-07-17

**Authors:** Marie Fält, Eskil Degsell, Petter Gustavsson, Ann-Christin von Vogelsang

**Affiliations:** 1 Department of Neurosurgery, Karolinska University Hospital, Stockholm, Sweden; 2 Neuro-Oncology Clinical Research, Innovation, Implementation and Collaboration, Karolinska University Hospital, Stockholm, Sweden; 3 Department of Microbiology, Tumor and Cell Biology, Karolinska Institutet, Stockholm, Sweden; 4 Division of Psychology, Department of Clinical Neuroscience, Karolinska Institutet, Stockholm, Sweden; 5 Department of Neurobiology, Care Sciences and Society, Karolinska Institutet, Stockholm, Sweden

**Keywords:** Delivery of healthcare, Informal caregiver, Quality improvement, Systematic review

## Abstract

**Background:**

Despite the substantial experience and knowledge generated daily by informal caregivers and their well-documented contribution to health outcomes, quality improvement efforts in healthcare continue to focus primarily on patients’ experiences. Yet patients’ perspectives differ in important ways from those of informal caregivers. The aim of the study was to explore how informal caregivers’ experiences are collected and used to inform QI initiatives in healthcare settings, rather than to examine caregivers’ experiences per se.

**Methods:**

A mixed-methods systematic review with a convergent segregated approach was conducted. Searches in four scientific databases (Medline, Web of Science, PsycInfo, and CINAHL) from inception to 20 May 2025 were conducted. The searches yielded 8,648 records, whereof 83 full texts were screened, and 13 studies met the inclusion criteria.

**Results:**

Experience-based co-design or its accelerated form were common methods for collecting experiential data. A wide range of interventions were implemented using these data, targeting improvements in clinical care, education, research, or system-level processes. Most studies focused on enhancing clinical care, including changes to care processes, improved interdisciplinary teamwork, and strengthened family-centred communication. System-level interventions addressed areas such as complaints and grievance reporting, and investigation of adverse events.

**Conclusion:**

Integrating the perspectives of informal caregivers into quality improvement initiatives has the potential to improve clinical care, strengthen medical and nursing education, inform clinical research, and enhance patient safety.

**Clinical trial number:**

Not applicable.

**Protocol registration:**

The study protocol was registered on PROSPERO before the commencement of the review (registration number CRD42023400597).

**Supplementary Information:**

The online version contains supplementary material available at 10.1186/s12912-026-05065-1.

## Background

Beyond their contribution to health outcomes, patients and their social networks accumulate extensive experiential knowledge through the management of daily life, both in the absence and presence of chronic illness. Over time, they become experts in their own or their significant others’ conditions [1]. Although research has historically focused on patients’ experiential knowledge, there is increasing recognition that self-management is rarely an individual endeavor; rather, it is often embedded within, and dependent on, the support offered by an individual’s social network [2]. Active social networks, including informal caregivers and compassionate communities play a key role in providing such support [3].

In cancer care, for example, competent social networks offer psychosocial support, physical assistance, and medical management [4], often acting as essential partners in an integral part of symptom management and even as primary decision-makers [5]. Moreover, in oncology an active and competent social network functions as an independent prognostic factor for overall survival in breast cancer [6], and may even contribute more to survival in glioblastoma in comparison to currently approved medical treatments [7]. In stroke care, informal caregivers are essential in providing the daily support required for recovery [8]. They are also a vital component in the care of patients with bipolar disorder [9].

Despite growing recognition of the critical role informal caregivers play in care coordination, continuity of care, and patient outcomes, healthcare quality measurement and improvement initiatives have historically focused primarily on patient-reported experience measures, placing comparatively less emphasis on caregiver-reported experiences [10]. Thus, quality improvement (QI) efforts in healthcare have largely continued to focus on patient-reported experiences. As a starting point for conducting this review, we examined the work by Gleeson et al. [11], who reviewed approaches to using patient experience data for QI in healthcare. Their analysis included 11 studies that used patient reported experience measures, primarily collected via surveys [11]. However, the perspectives of informal caregivers are distinct from those of patients, and both are essential for comprehensive QI. Therefore, this study aimed to explore how informal caregivers’ experiences are collected and used to inform QI initiatives in healthcare settings, rather than to examine caregivers’ experiences per se. Specifically, we addressed the following four research questions:

1. Which methods are used to collect informal caregivers’ experiences for QIs in healthcare?
2. Which QI interventions are informed by these experience data?
3. What are the effects of QI Interventions guided by informal caregivers’ experiences?
4. How do informal caregivers describe their experiences of participating in QI projects?

## Methods

### Study design and reporting

This mixed-methods systematic review had a convergent segregated approach [12]. The study protocol was registered in the International Prospective Register of Systematic Reviews (PROSPERO), with registration number CRD42023400597). The Preferred Reporting Items for Systematic Reviews and Meta-Analyses (PRISMA) guidelines were used to report the findings [13].

### Search strategy

The PICO (population/problem, intervention, comparison, outcome) framework was used to guide the database searches [14] as follows:

*Population*: Adults, informal caregivers who were actively providing care to patients with acute injuries/diseases or long-lasting diseases, or who were bereaved. An informal caregiver is defined as a person’s closest relation, not limited to those related by blood or marriage, and may also include extended family members or anyone significant to the person’s well-being, such as a spouse or a person in a similar relationship [15].

*Intervention*: QI interventions founded on informal caregivers’ experience data on all organizational levels in healthcare. For this review QIs are defined as interventions to improve the efficiency or effectiveness of a program, process or organization and may include reducing inefficiency, error and redundancy [16].

*Comparison*: Depending on included study design, the comparator included before - after intervention or intervention group - standard care. However, some studies such as observational studies or qualitative studies had no control groups.

*Outcome*: Informal caregivers’ experience data for QI interventions in healthcare, in terms of methods used, interventions and effects.

### Eligibility criteria

Studies were assessed eligible if they fulfilled the PICO and the following specified inclusion criteria:

1. Describes the collection and use of informal caregivers’ experience data for the explicit purpose of informing QI.
2. The experience data used for QI should be focused on healthcare improvement and not reported satisfaction, family caregiver burden or grief processes.
3. Describes QI actions undertaken in healthcare settings or by healthcare staff that were directly informed by informal caregivers’ experience data.
4. Includes measurement or recording of change through QI that was informed by informal caregivers’ experience data.

Quantitative (randomized controlled trials [RCT], observational studies), qualitative (interview studies, ethnographic observations) and mixed methods designs were considered eligible. Studies were excluded if published in other languages than English, or Scandinavian languages, and studies reporting duplicated results from one study/program, where all results could be retrieved from an included report.

A literature search was performed in four databases: Medline (Ovid), Web of Science (Clarivate Analytics), PsycInfo (EBSCO) and CINAHL (EBSCO). The search strategy was developed in Medline (Ovid) in collaboration with librarians at the Karolinska Institutet University Library. For each search concept Medical Subject Headings (MeSH-terms) and free text terms were identified. The search was then translated, in part using Polyglot Search Translator [17], into the other databases. No language restriction was applied, and databases were searched from inception. De-duplication was done using the method described by Bramer et al. [18]. One final, extra step was added to find duplicates by comparing DOIs.

After the original search was performed on 1 March 2021, the search was last updated on 20 May 2025 by a librarian, who reran the searches and deduplicated the results against previous records using Covidence [19].

**Fig. 1:**
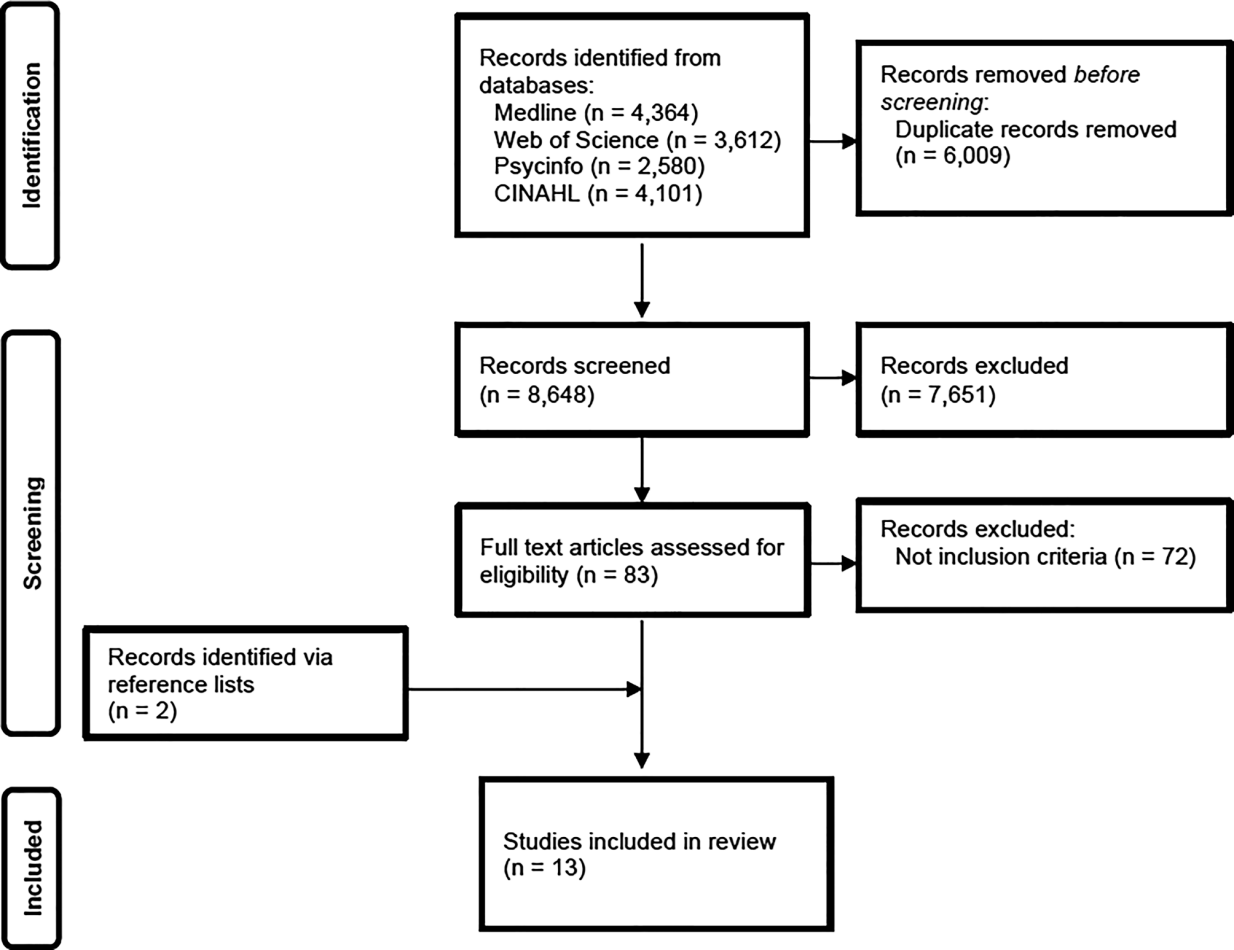
Flow diagram illustrating the identification of studies included in the review

Additionally, reference lists for included articles were screened to identify any studies left out from the database searches. Figure 1 shows a PRISMA flow chart of database searches and inclusion of studies.

The database search retrieved 14,657 records, and after removing duplicates, 8,648 titles remained. The search processes are reported per database in Supplementary file 1. The Rayyan web application [20] was used to support the review process with blinded individual selection of articles. Two reviewers screened all titles to assess whether they met the inclusion criteria. The abstracts of the studies identified as potentially eligible were independently reviewed in pairs by four reviewers. The same reviewer pairs also screened the reference lists of included articles to identify additional studies not captured through the database searches. Discrepancies regarding the inclusion of specific articles were resolved through discussion among all four reviewers, and the process was documented.

### Critical appraisal

To ensure consistency across studies, we selected a critical appraisal instrument that applies the same criteria regardless of research design. The tool, described by Rees et al. [21] and McDermott et al. [22], accommodates all study types and evaluates seven domains: theoretical framework, aim, context/setting, sample, methodology, validity and reliability of the data analysis, and the adequacy of original data provided to support interpretation. It also includes an overall assessment using four graded levels:

A – No or few flaws

B – Some flaws

C – Significant flaws, which may affect the validity of the findings

D – Untrustworthy findings/conclusions

The risk of bias/quality assessment was performed independently in pairs. Discrepancies were solved through discussions among all four reviewers.

### Data extraction

Data was extracted from the studies selected for inclusion independently in two stages. First, extraction was performed by two reviewers and thereafter verified by another reviewer that all relevant information was captured. Quantitative and qualitative components were extracted separately and simultaneously. The items extracted included sources of evidence (authors, title, year, and country), aim, methodology: design, setting and sample, data collection, data analysis. Further, key findings: interventions used, process of involving informal caregivers’ results of, and if available, informal caregivers’ experiences of participating in QI projects.

### Data analysis and synthesis of results

A convergent segregated approach was used, which included separate and simultaneous analysis and synthesis of quantitative and qualitative data [12]. Statistical pooling of quantitative data was not possible, and data was therefore summarized in tables. Qualitative data were synthesized using a thematic approach, whereby findings from the qualitative components were assembled, compared, and categorized based on similarities in meaning. These categories were subsequently aggregated into broader themes representing shared patterns across studies, which were then integrated with the quantitative findings in the final stage of the review.

## Results

### Study characteristics

Of the 13 articles and reports (published between 2002 and 2025) included in the final review, six were conducted in North America [23–28], six in the United Kingdom [29–34] and one in Norway [35]. All studies were undertaken within the context of healthcare services for physical illnesses. The settings included pediatric care [23, 25, 28, 30, 32], and adult general medical/surgical care [26, 35], stroke care [33], emergency care [29], intensive care [27], lung cancer care [31], palliative care [24], and care of older persons [34]. The number of informal caregivers included across studies ranged substantially, from *n* = 5 [29] to *n* = 2,148 [25]. The precise total number of informal caregivers included in the review could not be determined because several studies reported aggregated numbers for patients and informal caregivers or did not specify participant numbers.

Most studies employed a mixed-methods design [24, 28, 30, 32–34]. Two studies were process evaluations [31, 35] and four studies used experimental designs, including one RCT [23] and three quasi-experimental studies [25–27]. One mixed-methods study incorporated several sub-studies with distinct designs, i.e. qualitative descriptive, RCT, process evaluation and health economics components [34]. Most studies used at least two different data collection methods (but not always for gathering informal caregivers’ experience data), with interviews and observations being the most common combination [23, 29, 31, 33, 35]. Six studies collected questionnaire data, four in combination with other data sources [23, 25, 28, 31], and two as the sole method [27, 32]. Three studies used registry-based data, including medical records [24, 25], hospital incident reports [25] and registered complaints and grievances [26]. Table 1 summarizes methodological characteristics of the included studies.

**Table 1:** Methodological characteristics of included studies

Authors, Country	Aim	Study design, settings and samples	Data collection methods included in study	Data analysis
Blackwell et al. [29] UK	To critique the feasibility of using evidence-based co-design methodology as a QI intervention in complex healthcare settings	Mixed-methods design, Emergency Department in a teaching hospital. Interview samples: - Staff members (*n* = 79), - Patients (*n* = 6) - Family caregivers (*n* = 4) Co-design event sample: - Patient (*n* = 1), - Family caregiver (*n* = 1) - Staff members (*n* = 7) - Facilitator (*n* = 3) - Researcher (*n* = 2)	- Non-participant observations - Semi-structured interviews with staff members - Focus group interviews with staff members - Semi-structured interviews with palliative care patients or their family members	Thematic content analysis
Cox et al. [23] USA	To examine the impact of a family-centered rounds (FCR) checklist intervention, a checklist and associated provider training, on performance of FCR elements, family engagement, and patient safety	Randomized trial. Children’s hospital. Cluster randomization of four in-patient units: two general pediatric units, one pulmonary unit, one hematology/ oncology unit. - Two treatment arms (usual care/ intervention). Pre- and post-intervention samples: - Usual care admissions: Parents preintervention (*n* = 70) - Parents postintervention (*n* = 76) Intervention admissions: - Parents preintervention (*n* = 74) - Parents postintervention (*n* = 78)	- Study-specific survey - Video recorded observations of each FCR during the child’s stay - The Children’s Hospital Safety Climate Questionnaire filled in by the parent at hospital discharge	Descriptive and analytic statistics
Coy et al. [30] UK	To comprehensively explore emotional and experiential aspects of recovery after a child’s moderate burn injury	Mixed-methods design. Children’s Burn Centre at Bristol Royal Hospital for Children Interview sample: - Parents (*n* = 3) - Healthcare staff (*n* = 4) Co-design event sample: - Parents (*n* = 11) - Children (*n* = 12) - healthcare staff (*n* = 6)	- Semi-structured filmed interviews (parents) - Semi-structured audio-taped interviews (healthcare staff)	Filmed parent interviews: review, verification and editing by parents. Thematic content analysis of healthcare staff interview data
de Pins et al. [28] USA	To enhance post visit communication between providers and caregivers in a multidisciplinary pediatric neuromuscular program	Mixed-methods design. Pediatric tertiary care hospital. Survey sample: - Parents, *n* = 21 Interview sample: - Parents, *n* = 4	- Family Experience with Coordination of Care survey - In-depth interviews	Survey data: descriptive statistics. Analysis of interviews not described
Dahan et al. [32] Canada	To analyze activities involving resource parents and patients in a family partnership program	Mixed-methods design. Teaching hospital, Neonatal intensive care. - Healthcare staff (*n* = 27) - Resource parents (*n* = 28) - Resource patients (*n* = 2)	- Study-specific questionnaires with open-ended questions - Numerical data from quality improvement initiatives	Thematic content analysis, descriptive statistics
Jacobs et al. [24] USA	To evaluate a quality-improvement program for hospitalized dying patients	Mixed-methods design. Urban teaching hospital, palliative care - Physicians (*n* = 46) - Patients (*n* = 194) - Informal caregivers (*n* = 133)	Palliative care reports based on: - Medical records review - Family interviews by phone - Physician survey by phone	Descriptive and analytic statistics
Jones et al. [33] UK	To evaluate the feasibility and impact on patients, carers and staff co-producing and implementing interventions to increase supervised and independent therapeutic patient activity in acute stroke units	Mixed-methods design. Four stroke units, three at District general hospitals, and one at a city-center teaching hospital - Staff *n* = 130 - Patients *n* = 76 - Informal caregivers *n* = 47	- Semi-structured interviews - Observations - Behavioral mapping - Surveys - Process evaluation	Thematic analysis of interview and observational data. Descriptive statistics for survey data and behavioral mapping data
Khan et al. [25] North America	To determine whether medical errors, family experience, and communication processes improved after implementation of an intervention to standardize the structure of healthcare provider-family communication on family centered rounds	Quasi-experimental design (before/after intervention). In-patient pediatric medical units in one Canadian and six US teaching hospitals - Parents or caregivers (*n* = 2,148) - Nurses (*n* = 435) - Medical students (*n* = 203) - Physicians (*n* = 586)	Data on medical errors and adverse events collected through: - Review of medical records - Physician and nurse surveys - Hospital incident reports - Family safety interviews	Descriptive and analytic statistics
Kleinpell et al. [27] USA	To implement patient- and family-centered care engagement initiatives	Quasi-experimental design (before/after intervention. 63 pediatric and adult intensive care units from community and academic hospitals - Family members pre-intervention (*n* = 1,705) - Family members post-intervention (*n* = 825) - Healthcare staff pre-intervention (*n* = 2,942) - Healthcare staff post-intervention (*n* = 1,057)	- The family satisfaction with care in the ICU survey to collect family perceptions of care - A self-assessment inventory to collect ICU staff perceptions of family engagement and unit climate for supporting patient and family engagement	Descriptive and analytic statistics
Levin & Hopkins [26] USA	To create a streamlined patient complaint capture and resolution process to improve the capture of patient complaints and grievances from multiple parts of the organization and manage them in a centralized database	Quasi-experimental design (before/after intervention). Stanford Health Care (SHC), Not-for-profit teaching hospital, general medical surgical care. Numbers of patients/family members included not presented	Tracked patient/family complaints and grievances from the Patient Representative Department (PRD) data management system	Descriptive statistics
Locock et al. [31] UK	To use a national video and audio archive of patient experience narratives to develop, test and evaluate a rapid, patient-centred service improvement approach (‘accelerated experience-based co-design’ or AEBCD)	Longitudinal ethnographic process evaluation. Two NHS hospital trusts, intensive care and lung cancer services. - Clinical staff (*n* = 96) - Patients and family members (*n* = 63)	- Semi-structured interviews (staff) - Observations - Group interviews (patients) - Evaluation questionnaires (patients and staff) - Reflective diaries	- Qualitative framework analysis - Descriptive statistics - Cost analysis
Murray et al. [34] UK	To capture the experience of older patients and their families during the transition from hospital to home, and to identify opportunities for greater patient involvement in care	Mixed-methods design, six work packages (WPs): - WP 1 & 2: Qualitative methods - WP 3: Literature review, Delphi - WP 4: Co-design - WP 5: Cluster feasibility trial - WP 6: Cluster RCT, process evaluation, health economics. National Health Service acute hospitals trust, general practices, patient and carer homes. Informal caregivers involved in two WPs: WP 1: - Patients *n* = 32 - Informal caregivers *n* = 18 WP 4: - Patients and informal caregivers *n* = 25	WP 1: Semi-structured interviews and observations. WP 4: Semi-structured interviews and observations	WP 1: Thematic content analysis. WP 4: Iterative template approach, structured around the aims of the WP.
Wiig et al. [35] Norway	To explore regulatory inspectors’ experiences with a new method for next-of-kin involvement in investigation of adverse events causing patient death	Qualitative process evaluation. Two counties, general medical surgical care. Focus group sample: - Regulatory inspectors (*n* = 5). Observation sample: - Bereaved (*n* = 16)	- Focus group interviews (inspectors) - Observation of meetings	Thematic content analysis

### Methods used to collect informal caregivers’ experience data

A common method to collect experience data was experience-based co-design (EBCD), or an accelerated/abbreviated version (AEBCD) [29–31, 33]. Both the EBCD and AEBCD approaches involved creating trigger films, edited compilations of filmed interviews or narrative excerpts from multiple individuals, which highlighted key touchpoints and served as tools for capturing experiences, facilitating reflective learning, and fostering emotional connection among patients, families, and healthcare staff. The process was accelerated either by transferring QI initiatives developed in one unit to a similar unit providing the same type of care in another department or another hospital [33], or by using an existing archive of filmed interviews for the construction of trigger films [31]. Other methods used to collect experience data were interviews [23, 24, 28, 30, 34], questionnaires [27, 28], and observations of meetings between inspectors (i.e., a medical doctor and a legal practitioner involved in regulatory investigations) and bereaved family members in investigations of adverse events resulting in patient death, with the aim of integrating informal caregivers’ experiential insights into system-level investigations [35]. Further methods included documented complaints from hospital patients and their families [26], as well as formulated programs including partnership between families and healthcare staff [25, 32].

### QI interventions using informal caregivers’ experience data in healthcare

A wide range of interventions were based on experience data, some studies described few and detailed interventions such as using a family-centred checklist [23], while other studies briefly described many interventions by organizational level [31] or mentioned hundreds of interventions [32]. The interventions could be divided into interventions to improve clinical care, education, research, and system improvements. Most studies described interventions focused to improve clinical care [23, 25, 27, 31, 34]. One study described interventions to both improve clinical care and education for healthcare staff [24], while another study also outlined research interventions, other than clinical care and education interventions [32]. Two studies included interventions for system improvements, for complaints and grievance reporting [26], and for investigations of adverse events [35]. The content of the clinical care interventions varied widely, and included, for instance, changed care processes [29, 31], improved interdisciplinary teamwork [29] and enhanced family-centred care/communication [23, 25, 27].

### Effects of QI interventions guided by informal caregivers’ experience data

The effects varied according to the study context, aims and interventions implemented. Improvements were noted in clinical care and care pathways [24, 27, 29, 31, 33, 34], in information materials and communication processes [23, 28, 30, 31, 33], and in care environments [33]. Enhancements in patient safety were also reported: quality of adverse events investigations improved [35], and the number of preventable adverse events decreased [25]. In addition, the number of captured complaints increased [26]. Table 2 summarizes the methods used to collect experience data, the corresponding QI interventions, and their effects.

**Table 2:** Methods used to collect experience data, QI interventions and effects of QIs

Study	Methods used to collect informal caregivers’ experience data	QI interventions using informal caregivers’ experiences data in healthcare	Effects of QI interventions guided by informal caregivers’ experiences data
Blackwell et al. [29]	EBCD-process based on filmed semi-structured interviews edited to a trigger film and co-design events.	- Increased interdisciplinary teamwork - Changed care processes	- Collation and dissemination of palliative care services and support information - Improved pathway through the emergency department experience for palliative care patients and their families
Cox et al. [23]	Stimulated recall interviews	A family-centered rounds checklist including prioritized activities: - Assessments - Care plan - Discharge goals - Communication - Information gathering and giving - Perceptions of safety - Handoffs and transitions	Improved information exchange: - The families were asked more questions during family-centered rounds - The healthcare team were asked more questions during family-centered rounds - Healthcare read back orders during family-centered rounds increased
Coy et al. [30]	AEBCD-process based on semi-structured interviews and construction of a trigger film and a focus event.	- Facilitated collaboration between parents - Information material (i.e., photo series of different degrees of burn injury and how they progress) - Information provision from healthcare staff	- Provision of appropriate information - Improved communication between the service, patients, and their families - Increased healthcare staff awareness of the emotional aspects of burn injuries
de Pins et al. [28]	Questionnaire and in-depth interviews	- Creation of template text for standardized patient information - Review of critical elements with providers, and individualized feedback	- Increased completion of after-visit summaries (AVS, an overview document for care guidance, medication list, contact information, future appointments) - AVSs more concise and understandable
Dahan et al. [32]	Family partnership program where resource parents were teamed up with healthcare providers and were integrated to different degrees in various initiatives and activities.	A total of 653 activities related to clinical care or administration (*n* = 413), education (*n* = 31) and research (*n* = 209), from low risk to complex activities	- Transfer of experiences and parental perspective into clinical care, administration, medical education and research - Improved clinical care - Improved communication - Improved research projects
Jacobs et al. [24]	Structured telephone interviews	- Palliative care social worker consultations - Educational programs for healthcare staff - Palliative care reports (providing direct evaluation feedback from physicians to physicians)	Improved clinical care (i.e., management of respiratory symptoms)
Jones et al. [33]	EBCD and AEBCD processes using non-participant observations, filmed interviews edited to trigger films and co-design meetings	- Environmental improvements - Increased activity opportunities - Increased communication opportunities	Mixed results across the four units - Most patients reported having sufficient number of activities post interventions - Improved information provision
Khan et al. [25]	An iterative consensus building process through a series of teleconferences, meetings, informed by a review of literature and previous research.	A co-produced intervention based on structured communication which included: - Changes to verbal communication on rounds - Completion of round reports - Structured observations with assessment and feedback	- The overall rate of medical errors was unchanged - Preventable adverse events decreased (*p* = 0.01) - Non-preventable adverse events decreased (*p* = 0.003) - Family-centered rounds occurred more frequently (*p* = 0.02) - Increased family engagement (*p* = 0.04) - Increased nurse engagement (*p* = 0.03)
Kleinpell et al. [27]	A national collaborative using a nationwide patient and family advisory group, who developed a manual for collaborative planning. Experience data collected from and pre- and post-intervention surveys.	Each ICU implemented their self-proposed local engagement interventions. Typical interventions were: - Family care conferences - Family-centered rounds - Patient/family diaries	In comparison with pre-intervention data: - Families felt more included in the decision-making process (*p* = 0.014) - Families felt more supported during the decision-making process (*p* = 0.002) - Families perceived more control over the care of the family member (*p* = 0.011)
Levin & Hopkins [26]	Complaints and grievances from hospital patients and their families	The intervention constituted five areas: - Leadership engagement - Increased capture of patient complaints Centralized data and reporting - Improved response time - Improved service	- Complaints captured increased - Increased identification of physicians associated with a complaint - Improved physician interaction with patients
Locock et al. [31]	AEBCD-process based on an existing archive of filmed narrative interviews and construction of new trigger films.	An intervention including a total of 48 improvement activities: - Small scale changes - Process redesign at the team level - Process redesign between services - Process redesign between organizations	- Improved nursing care - Redesigned patient information processes and information material - Improved patient transfers - Improved access to out-hours services
Murray et al. [34]	Semi-structured interviews and observations of care	‘Your care needs you’ intervention, including: a booklet, a film, a ‘Patient advice to help you at home’ sheet	- A reduction in total re-admissions of 13% over 90 days - At 30 days post discharge significantly less adverse events (such as falls) were reported
Wiig et al. [35]	Observations of inspectors’ meetings with bereaved who had lost a family member in an adverse event.	Bereaved family members’ involvement in investigation of adverse events	- Improved investigation quality (i.e., bereaved family members was a key source of information, contributed to a more holistic view of the adverse event and the involved stakeholders) - Emotionally challenging for inspectors - Increased workload for inspectors

### Informal caregivers’ experiences of participating in quality improvement projects

Three studies [30–32] described informal caregivers’ experiences of participating in QI projects, of which most described positive experiences. Informal caregivers valued meeting others with similar experiences [30, 31], and appreciated opportunities to share ideas with healthcare professionals [30]. They felt listened to, believed their perspectives were taken seriously, that perceived themselves as equal partners whose contributions led to meaningful improvements in care [31, 32]. Participation was also described as a meaning-making process that helped informal caregivers make sense of previous experiences and was recognized as a way to give back and support others [32]. However, involvement also brought challenges, including the resurfacing of difficult memories and the re-experiencing of traumatic events [32].

### Quality appraisal of included studies

Most of the included studies were assessed to have few or some methodological flaws. The detailed quality appraisal is presented in Table 3.

**Table 3:** Quality appraisal for included studies

Assessment areas Study	Theoretical framework	Aims	Context/setting	Sample	Methodology	Validity and reliability of data analysis	Sufficient amount of data	Overall assessment
Blackwell et al. [29]	B	B	B	C	B	B	B	B
Cox et al. [23]	A	A	A	B	B	A	B	A
Coy et al. [30]	A	A	A	B	C	B	B	B
Dahan et al.[32]	C	B	B	B	C	B	B	C
de Pins et al. [28]	B	A	B	C	C	C	B	C
Jacobs et al. [24]	B	C	B	B	C	D	C	C
Jones et al. [33]	A	A	A	B	A	B	A	A
Khan et al.[25]	A	A	A	A	A	A	A	A
Kleinpell et al.[27]	B	C	C	B	C	C	D	D
Levin & Hopkins. [26]	B	C	B	C	C	B	A	C
Locock et al.[31]	A	A	A	B	A	A	B	A
Murray et al. [34]	A	A	A	C	A	A	A	A
Wiig et al. [35]	C	B	C	C	C	B	C	C

## Discussion

This review aimed to explore how informal caregivers’ experiences are collected and used to inform quality improvement (QI) initiatives in healthcare, rather than to examine caregivers’ experiences per se. The review demonstrates that, although informal caregivers’ experiential knowledge plays a crucial role in shaping care delivery, relatively few studies have systematically incorporated these perspectives into QI efforts. Only 13 articles met the inclusion criteria, spanning diverse healthcare contexts but with a noticeable concentration in pediatric care. Across these studies, experience data were collected using a range of methods, from structured approaches such as EBCD and AEBCD to interviews, questionnaires, complaints data, and observational methods, highlighting the methodological heterogeneity in current practice. Despite this variation, caregivers’ experiences consistently informed meaningful improvements in clinical care, communication processes, care environments, and patient safety. Before starting this review, we examined the work of Gleeson et al. [11], who explored how patients’ experiences are collected, communicated, and utilized to inform QI in healthcare. In comparison with our findings, clear differences emerged in both the methods used to collect experience data, and the types of QIs implemented. This underscores the importance of incorporating not only the patient’s perspective but also the perspectives of informal caregivers, perspectives that are distinct and not interchangeable.

There was considerable variation in the types of interventions described in the included studies, as well as how they were implemented and evaluated. Some investigations focused on a single, targeted intervention, whereas others introduced large and complex sets of changes. Through studies, interventions could be grouped into four categories: improvements to clinical care, education, research, and system-level processes. Most interventions aimed to enhance clinical care [24, 27, 29, 31, 33, 34], with several resulting in permanent changes to daily routines that enabled the systematic use of experience-based insights. The effects of these interventions varied according to study design and complexity; some achieved small, localized improvements [28, 30, 35], while others led to changes across multiple sites [25, 27]. Descriptions of informal caregivers’ experiences in QI projects were limited, however, the experiences reported were largely positive and meaningful, regardless of whether the intervention constituted a small-scale initiative or a more extensive organizational change at the system level.

A commonly used method for collecting experience data was EBCD, or AEBCD [29–31, 33]. EBCD, first piloted in 2005 in head and neck cancer care [36], was initially developed to engage patients and users through interviews and surveys to identify strengths and areas for improvement in care [37]. Over time, it evolved into a more participatory and continuous model in which stakeholders jointly identify, implement, and evaluate improvements in healthcare services [38]. EBCD is grounded in the principles of partnership, treating staff, patients, and other users as equal contributors guided by openness, respect, and empathy [39]. Despite its strengths, EBCD is resource-intensive; a full process typically requires 9–12 months, and implementation barriers such as insufficient funding, limited managerial support, staff turnover, and logistical constraints are common [36]. Nonetheless, involving informal caregivers in QI initiatives appears to have benefits beyond improving services. Studies reporting their experiences indicate strong altruistic motivations, such as a desire to help others [32], as well as positive perceptions of being heard and being treated as equal partners, regardless of whether the project used EBCD [31] or other participatory approaches [32]. These findings align with a systematic review of EBCD studies [36], in which participants similarly reported positive experiences and perceived the power balance as more equal. Co-production through co-design thus offers notable opportunities and challenges. It allows patients, informal caregivers, and healthcare professionals to collaborate under more equitable conditions, fostering inclusion, solidarity, and the generation of new insights. At the same time, its open and flexible nature risks being reduced to a procedural trend rather than a meaningful approach if not implemented thoughtfully. Co-design is most effective when approached as an exploratory social process that generates new perspectives, rather than as a tool used solely to achieve predetermined outcomes [40].

Using the experiences of informal caregivers for QI in healthcare requires significant commitment to be effective. If such work is discontinued, the data can still be utilized for QI to support the next generation of patients and informal caregivers [41], even beyond the healthcare system [42]. If the collected data can be applied in multiple contexts, the overall impact is likely to be greater. With increased utilization, such work may even shift from being project-based to becoming routine practice [43].

Many of the studies included in this review were project-based. However, some studies [25, 26] demonstrated approaches in which the collection and use of experiential data were embedded into routine practice, forming the foundation for large-scale and continuous learning systems. This aligns with the concept of learning health system (LHS), which describes a team, provider or network of providers that, together with a community of stakeholders, develops the capacity to learn from its own delivery of routine care and improve continuously as a result. At its core, an LHS consists of the structures, processes, and resources that enable ongoing learning and improvement of services [44]. The development of such continuous learning systems, in which all perspectives, including those of informal caregivers are systematically incorporated, may represent an important direction for the future of quality improvement in healthcare.

This study has several limitations that should be acknowledged. Firstly, the quality appraisal of the 13 included studies showed that seven [23, 25, 29–31, 33, 34] had few or some flaws and six [24, 26, 28, 32, 35] had significant flaws and lacked adequate descriptions of used research methods. Moreover, our database search specifically targeted studies that reported the full process; from collecting informal caregivers’ experience data, to designing interventions, and to describing or evaluating their effects. Only a small number of studies met these criteria. It is likely that additional research using similar processes exists but was not captured because the different stages of the work were either not published or were published separately, making it difficult to identify them as part of a comprehensive QI process.

This is a relatively new field of research, with the earliest study published in 2002 [24] and the latest 2025 [34], with the vast majority of studies appearing between 2017 and 2021. Several database records describing simplified versions of EBCD were identified; however, these did not meet the inclusion criteria and were therefore excluded. It is possible that additional evidence exists but remains unpublished, as many interventions conducted within healthcare settings where findings are retained locally rather than disseminated through academic publications. The prominence of EBCD and AEBCD among the included studies may indicate a growing interest in the use of narrative-based experiential data to inform quality development.

In conclusion, research on using informal caregivers’ experience data to inform quality improvements in healthcare remains limited. EBCD/AEBCD represents resource-intensive yet effective processes for co-creation in healthcare, placing narratives on experiences at the center of shared understanding, reflection and learning. The QI interventions identified in this review were implemented at multiple organizational levels and targeted diverse areas, including clinical care, education, clinical research, and administrative systems. The observed effects were similarly varied and reflected the heterogeneity and complexity of the study designs and methods used. Thus, integrating the perspectives of informal caregivers into quality improvement initiatives has the potential to enhance clinical care, strengthen medical and nursing education, inform clinical research and improve patient safety.

## Electronic supplementary material

Below is the link to the electronic supplementary material.

Supplementary Material 1: Search strategies reported per database.

## Data Availability

All relevant data are within the manuscript and its Supporting information files.

## Abbreviations

AEBCD: Accelerated/abbreviated Experience-Based Co-Design
EBCD: Experience-Based Co-Design
LHS: Learning Health System
PRISMA: Preferred Reporting Items for Systematic Reviews and Meta-Analyses
PROSPERO: Prospective Register of Systematic Reviews
QI: Quality Improvement
RCT: Randomized Controlled Trial
