# Supplementary material for: Using informal caregivers’ experience data to inform quality improvement in healthcare settings: a mixed-methods systematic review
Source: BMC Nurs. 2026 Jul 17;25:637. doi: 10.1186/s12912-026-05065-1 (PMC13379961; doi:10.1186/s12912-026-05065-1)
Supplement: Supplementary file 1 — Supplementary Material 1: Search strategies reported per database. [file 12912_2026_5065_MOESM1_ESM.docx]

1. Medline

| Interface: **Ovid MEDLINE(R) ALL** content coverage from 1946  Date of Search: May 20, 2025  Number of hits: 4,364  Comment: In Ovid, two or more words are automatically searched as phrases; i.e. no quotation marks are needed | Field labels   - exp/ = exploded MeSH term - / = non exploded MeSH term - .ti,ab,kf. = title, abstract and author keywords - adjx = within x words, regardless of order - * = truncation of word for alternate endings - ? = 0-1 letter/number - # = 1 letter/number |
| --- | --- |
| Database(s): **Ovid MEDLINE(R) ALL**1946 to May 19, 2025 Search Strategy:   \| **#** \| **Searches** \| **Results** \| \| --- \| --- \| --- \| \| 1 \| ((bereave* or caregiver* or family or families or "next of kin" or relative*) adj2 (experience* or perception* or perspective* or view* or satisfaction or involve* or engage* or engaging or feedback)).ti,ab,kf. \| 46087 \| \| 2 \| (Experience-based Co-design and (caregiver* or family or families or "next of kin" or relative*)).ti,ab,kf. \| 86 \| \| 3 \| Bereaved Family Survey.ti,ab,kf. \| 43 \| \| 4 \| 1 or 2 or 3 \| 46178 \| \| 5 \| Quality of Health Care/ \| 79535 \| \| 6 \| exp Quality Improvement/ \| 37506 \| \| 7 \| exp Quality Assurance, Health Care/ \| 361971 \| \| 8 \| exp Quality Indicators, Health Care/ \| 26230 \| \| 9 \| Health Plan Implementation/ \| 6698 \| \| 10 \| Organizational Innovation/ \| 25581 \| \| 11 \| (quality adj3 (assuranc* or enhanc* or improv* or indicator* or raise)).ti,ab,kf. \| 324793 \| \| 12 \| (quality adj2 (care or healthcare)).ti,ab,kf. \| 98201 \| \| 13 \| (organi#ational innovation* or health plan implement*).ti,ab,kf. \| 622 \| \| 14 \| or/5-13 \| 803534 \| \| 15 \| 4 and 14 \| 4364 \| | |

2. Web of Science Core Collection

| Interface: **Clarivate Analytics**  Editions and content coverage years= A&HCI - 1975 , ESCI -2019 , SCI-EXPANDED - 1945 , SSCI - 1945  Date of Search: May 20, 2025  Number of hits: 3,612 | Field labels   - TS/Topic = title, abstract, author keywords and Keywords Plus - TI= title - AB = abstract - AK = author keywords - NEAR/x = within x words, regardless of order - * = truncation of word for alternate endings - $ = 0-1 letter/number - ? = 1 letter/number   Note: the *Exact search*-function was used for all the searches |
| --- | --- |
| \| **#** \| **Search Query** \| **Results** \| \| --- \| --- \| --- \| \| 1 \| TS=((bereave* or caregiver* or family or families or "next of kin" or relative*) NEAR/1 (experience* or perception* or perspective* or view* or satisfaction or involve* or engage* or engaging or feedback) ) \| 65240 \| \| 2 \| TS=("Experience-based Co-design") AND TS=(caregiver* or family or families or "next of kin" or relative*) \| 84 \| \| 3 \| TS=("Bereaved Family Survey") \| 50 \| \| 4 \| #1 OR #2 OR #3 \| 65337 \| \| 5 \| TS=(quality NEAR/2 (assuranc* or enhanc* or improv* or indicator* or raise)) \| 523434 \| \| 6 \| TS=(quality NEAR/1 (care or healthcare)) \| 98991 \| \| 7 \| TS=(("organi#ational innovation*" or "health plan implement*")) \| 230 \| \| 8 \| #5 OR #6 OR #7 \| 588069 \| \| 9 \| #4 AND #8 \| 3612 \| | |

3. PsycINFO

| Interface: **EBSCOhost** - conctent coverage from 1806  Date of Search: May 20, 2025  Number of hits: 2,580 | Field labels   - DE = subject heading - TI = title - AB = abstract - KW = author keywords - XB = title, abstract - Nx = within x words, regardless of order - * = truncation of word for alternate endings - # = 0-1 letter/number - ? = 1 letter/number   Note: sometimes “quotation marks” are needed for single search terms to avoid automatic term mapping (lemmatization) |
| --- | --- |
| \| **#** \| **Query** \| **Results** \| \| --- \| --- \| --- \| \| S10 \| S4 AND S9 \| 2,580 \| \| S9 \| S5 OR S6 OR S7 OR S8 \| 107,900 \| \| S8 \| TI ( ("organi?ational innovation*" OR "health plan implement*" ) ) OR AB ( ("organi?ational innovation*" OR "health plan implement*" ) ) OR KW ( ("organi?ational innovation*" OR "health plan implement*" ) ) OR TM ( ("organi?ational innovation*" OR "health plan implement*" ) ) \| 833 \| \| S7 \| TI ( (quality N2 (care OR healthcare )) ) OR AB ( (quality N2 (care OR healthcare )) ) OR KW ( (quality N2 (care OR healthcare )) ) OR TM ( (quality N2 (care OR healthcare )) ) \| 29,586 \| \| S6 \| TI ( (quality N3 (assuranc* OR enhanc* OR improv* OR indicator* OR raise )) ) OR AB ( (quality N3 (assuranc* OR enhanc* OR improv* OR indicator* OR raise )) ) OR KW ( (quality N3 (assuranc* OR enhanc* OR improv* OR indicator* OR raise )) ) OR TM ( (quality N3 (assuranc* OR enhanc* OR improv* OR indicator* OR raise )) ) \| 56,302 \| \| S5 \| (DE "Quality of Care" OR DE "Quality Control" OR DE "Innovation" OR DE "Quality of Services") \| 46,822 \| \| S4 \| S1 OR S2 OR S3 \| 57,195 \| \| S3 \| TI "Bereaved Family Survey" OR AB "Bereaved Family Survey" OR KW "Bereaved Family Survey" OR TM "Bereaved Family Survey" \| 30 \| \| S2 \| TI ( ("Experience-based Co-design" AND (caregiver* OR family OR families OR "next of kin" OR relative* )) ) OR AB ( ("Experience-based Co-design" AND (caregiver* OR family OR families OR "next of kin" OR relative* )) ) OR KW ( ("Experience-based Co-design" AND (caregiver* OR family OR families OR "next of kin" OR relative* )) ) OR TM ( ("Experience-based Co-design" AND (caregiver* OR family OR families OR "next of kin" OR relative* )) ) \| 19 \| \| S1 \| TI ( ((bereave* OR caregiver* OR family OR families OR "next of kin" OR relative* ) N2 (experience* OR perception* OR perspective* OR view* OR satisfaction OR involve* OR engage* OR engaging OR feedback)) ) OR AB ( ((bereave* OR caregiver* OR family OR families OR "next of kin" OR relative* ) N2 (experience* OR perception* OR perspective* OR view* OR satisfaction OR involve* OR engage* OR engaging OR feedback)) ) OR KW ( ((bereave* OR caregiver* OR family OR families OR "next of kin" OR relative* ) N2 (experience* OR perception* OR perspective* OR view* OR satisfaction OR involve* OR engage* OR engaging OR feedback)) ) OR TM ( ((bereave* OR caregiver* OR family OR families OR "next of kin" OR relative* ) N2 (experience* OR perception* OR perspective* OR view* OR satisfaction OR involve* OR engage* OR engaging OR feedback)) ) \| 57,168 \| | |

4. CINAHL

| Interface: **EBSCOhost** - content coverage from 1981  Date of Search: May 20, 2025  Number of hits: 4,101 | Field labels   - MH+ = exploded CINAHL Heading - MH = non exploded CINAHL Heading - TI = title - AB = abstract - XB = title, abstract - Nx = within x words, regardless of order - * = truncation of word for alternate endings - # = 0-1 letter/number - ? = 1 letter/number   Note: sometimes “quotation marks” are needed for single search terms to avoid automatic term mapping (lemmatization) |
| --- | --- |
| \| **#** \| **Query** \| **Results** \| \| --- \| --- \| --- \| \| S13 \| S4 AND S12 \| 4,101 \| \| S12 \| S5 OR S6 OR S7 OR S8 OR S9 OR S10 OR S11 \| 438,347 \| \| S11 \| TI ( "organi#ational innovation*" or "health plan implement*" ) OR AB ( "organi#ational innovation*" or "health plan implement*" ) \| 159 \| \| S10 \| TI ( (quality N1 (care or healthcare)) ) OR AB ( (quality N1 (care or healthcare)) ) \| 55,790 \| \| S9 \| TI ( (quality N2 (assuranc* or enhanc* or improv* or indicator* or raise)) ) OR AB ( (quality N2 (assuranc* or enhanc* or improv* or indicator* or raise)) ) \| 111,650 \| \| S8 \| (MH "Quality Management, Organizational") \| 1,476 \| \| S7 \| (MH "Program Implementation") \| 41,469 \| \| S6 \| (MH "Quality Assurance+") \| 251,456 \| \| S5 \| (MH "Quality of Health Care") \| 88,240 \| \| S4 \| S1 OR S2 OR S3 \| 28,035 \| \| S3 \| TI “Bereaved Family Survey” OR AB “Bereaved Family Survey” \| 36 \| \| S2 \| TI ( “Experience-based Co-design” AND (caregiver* or family or families or "next of kin" or relative*) ) OR AB ( “Experience-based Co-design” AND (caregiver* or family or families or "next of kin" or relative*) ) \| 40 \| \| S1 \| TI ( (bereave* or caregiver* or family or families or "next of kin" or relative*) N1 (experience* or perception* or perspective* or view* or satisfaction or involve* or engage* or engaging or feedback) ) OR AB ( (bereave* or caregiver* or family or families or "next of kin" or relative*) N1 (experience* or perception* or perspective* or view* or satisfaction or involve* or engage* or engaging or feedback) ) \| 27,980 \| | |
